# Tumor-Educated Neutrophils Activate Mesenchymal Stem Cells to Promote Gastric Cancer Growth and Metastasis

**DOI:** 10.3389/fcell.2020.00788

**Published:** 2020-08-13

**Authors:** Jiahui Zhang, Cheng Ji, Wei Li, Zheying Mao, Yinghong Shi, Hui Shi, Runbi Ji, Hui Qian, Wenrong Xu, Xu Zhang

**Affiliations:** ^1^Jiangsu Key Laboratory of Medical Science and Laboratory Medicine, School of Medicine, Jiangsu University, Zhenjiang, China; ^2^Center of Research Laboratory, The First People’s Hospital of Lianyungang, Lianyungang, China; ^3^Department of Clinical Laboratory Medicine, The Affiliated People’s Hospital of Jiangsu University, Zhenjiang, China

**Keywords:** neutrophils, mesenchymal stem cells, cancer-associated fibroblasts, gastric cancer, progression

## Abstract

In response to tumor signals, mesenchymal stem cells (MSCs) are recruited to tumor sites and activated to promote tumor progression. Emerging evidences suggest that in addition to tumor cells, non-tumor cells in tumor microenvironment could also interact with MSCs to regulate their phenotype and function. However, the mechanism for MSCs regulation in gastric cancer has not been fully understood. In this study, we reported that tumor-educated neutrophils (TENs) induced the transformation of MSCs into cancer-associated fibroblasts (CAFs) which in turn remarkably facilitated gastric cancer growth and metastasis. Mechanistic study showed that TENs exerted their effects by secreting inflammatory factors including IL-17, IL-23 and TNF-α, which triggered the activation of AKT and p38 pathways in MSCs. Pre-treatment with neutralizing antibodies to these inflammatory factors or pathway inhibitors reversed TENs-induced transformation of MSCs to CAFs. Taken together, these data suggest that TENs promote gastric cancer progression through the regulation of MSCs/CAFs transformation.

## Introduction

Gastric cancer (GC) is one of the most common malignant tumors worldwide. Although the recent advances in curative resection and targeted therapy, the overall survival rate of patients with GC is still poor with a 5-year survival rate of 20–40% (Bray et al., 2018). The mechanisms for the pathogenesis of GC have not been fully understood. Intriguingly, recent studies demonstrate that the interactions between GC cells, immune cells, and stromal cells orchestrate a unique microenvironment that promotes tumor growth, metastasis, therapy resistance, and recurrence (Hanahan and Weinberg, 2011; Quail and Joyce, 2013).

Neutrophils are the first defense against infection and tissue damage. The studies over the past decade have revealed an important role of neutrophils in the pathogenesis of many cancers (Swierczak et al., 2015; Liang and Ferrara, 2016; Powell and Huttenlocher, 2016). Neutrophils infiltrating within tumor stroma were educated by signals from tumors to promote tumor growth and metastasis, enhance angiogenesis, and mediate immunosuppression through multiple mechanisms (Houghton et al., 2010; Antonio et al., 2015; Coffelt et al., 2015; Wculek and Malanchi, 2015; Szczerba et al., 2019; Wisdom et al., 2019). Moreover, elevated neutrophil to lymphocyte ratio (NLR) in cancer patients has been linked to poor prognosis (Shen et al., 2014). Targeting neutrophils to inhibit their pro-tumor function has shown promising therapeutic effects in animal models (Nywening et al., 2018). Therefore, better understanding of the roles of neutrophils in cancer will help develop new strategies for cancer therapy.

Non-tumor cells within tumor microenvironment (TME) interact with each other and form an intricate network that is involved in tumor development and progression. The previous studies have shown that cancer-associated fibroblasts (CAFs) facilitate cancer growth and metastasis in various cancers including GC (Chen and Song, 2019; Kobayashi et al., 2019). Further studies suggest that mesenchymal stem cells (MSCs) are one of the major sources of CAFs (Timaner et al., 2020). MSCs are stem cells with self-renewal and multi-differentiation abilities and they have shown tumor tropism to participate in the formation of tumor stroma (Quante et al., 2011). MSCs-like cells have been isolated from tumor tissues of GC patients and these cells display CAFs phenotypes with strong pro-tumor activities (Cao et al., 2009; Wang et al., 2014). MSCs can be activated by various tumor signals to present CAFs phenotype and function (Ridge et al., 2017). However, the mechanisms for MSCs transformation to CAFs in GC still have not been well characterized.

In this study, we reported that tumor-educated neutrophils (TENs) could mediate the transformation of MSCs to CAFs by secreting inflammatory factors. MSCs activated by TENs promoted the proliferation, migration and invasion of GC cells *in vitro* and accelerated GC growth and metastasis *in vivo*. The induction of MSCs/CAFs transformation represents a new mechanism for the pro-tumor roles of neutrophils in cancer.

## Materials and Methods

### Cell Culture

Mesenchymal stem cells were isolated from human umbilical cord and characterized as previously described (Cao et al., 2009; Wang et al., 2014). Cells at passage 3–5 were used for studies. The experimental protocol was approved by the Ethics Committee of Jiangsu University (2014280). Human GC cell lines SGC-7901 and BGC-823 were purchased from the Institute of Biochemistry and Cell Biology at the Chinese Academy of Sciences (Shanghai, China). Cells were cultured in Dulbecco’s modified Eagle’s medium (DMEM, Gibco; Thermo Fisher Scientific, Waltham, MA, United States) with 10% fetal bovine serum (FBS; Invitrogen, Carlsbad, CA, United States).

### Isolation of Human Neutrophils

Peripheral blood samples were collected from healthy volunteers and the study was approved by the ethics committee of Jiangsu University (2014280). Neutrophils were isolated by using Polymorphprep (Axis-Shield Po CAS, Norway) as previously described (Zhu et al., 2014). RBCs were lysed using hypotonic lysing procedure. The purity of neutrophils was 98% after this procedure. Neutrophils (1 × 10^6^) were seeded in RPMI 1640 medium (Invitrogen, United States) containing 10% (v/v) FBS and 1% penicillin/streptomycin.

### Collection of Conditioned Medium (Cm)

Firstly, GC cells were cultured in DMEM with 10% FBS. When reached 80%, cells were changed to new serum-free medium. After 48 h of incubation, the conditioned medium of gastric cancer cells (GC-CM) was collected and centrifuged to remove cell debris. The CM was stored in −80°C refrigerator until use. Secondly, neutrophils were treated with GC-CM (1:1 ratio) for 24 h. Then, neutrophils were collected, washed with PBS (to remove factors present in GC-CM), and re-seeded in new serum-free medium for 48 h. The CM of tumor-educated neutrophils (TEN-CM) was collected, centrifuged to remove cell debris and stored in −80°C refrigerator until use. Finally, MSCs were co-cultured with TEN-CM (1:1 ratio) for 24 h. Then, MSCs were collected, washed with PBS (to remove factors present in TEN-CM), and re-seeded in new serum-free medium for 48 h. The CM of TEN-CM-primed MSCs was collected, centrifuged to remove cell debris and stored in −80°C refrigerator until use.

### Cell Colony Formation Assay

Human GC cells were plated in 6-well plates at 800 cells per well and allowed to attach overnight. The cells were then treated with CM from TENs-activated MSCs for 24 h. Then, the CM was changed and added new culture medium for the next 7 days. Finally, the cells were fixed in 4% paraformaldehyde, stained with crystal violet, and photographed under a microscope. The experiments were performed in triplicate for each group.

### Cell Proliferation Assay

The proliferative abilities of GC cells and MSCs were determined by CCK8 (Vazyme, Nanjing, China) assay according to the manufacture’s instruction. Cells were seeded in 96-well plates at 3,000 cells per well and allowed to attach overnight at 37°C in 5% CO_2_. Then, different CMs were added into the culture plate and cells were treated for different times. The culture medium was discarded and CCK8 was added into each well for the last 4 h. The absorbances of each well were read at 450 nm using an enzyme-linked immunosorbent plate assay reader (FLX800; BioTek Instruments, Winooski, VT, United States). The experiments were performed in triplicate for each group.

### Cell Migration and Invasion Assays

Cell migration and invasion assays were tested in transwell chemotaxis chambers (Corning, Union City, CA, United States). Briefly, an appropriate 50 microliters of Matrigel (1:4 dilution, BD Biosciences) was added into the upper chamber of the transwell plates for the invasion assay, while the plates without Matrigel in the upper chamber were used for the migration assay. Cells (3–5 × 10^4^) treated with different CMs for 24 h were seeded in the upper chamber in serum-free medium. Then, 600 μL of complete medium was added to the lower chamber and the cells were incubated for 24–48 h at 37°C in 5% CO_2_. Cells which remained on the top side of the membrane were wiped off with cotton swabs. The migrated or invaded cells on the bottom side of the membrane were fixed in 4% paraformaldehyde and stained with crystal violet. The number of migrated or invaded cells was counted under a microscope. At least six fields from each group were selected.

### Real-Time Quantitative PCR

Total RNA was extracted from cells using Trizol reagent (Thermo Fisher Scientific, United States) according to the manufacturer’s instructions. RNA (1 μg) was reverse transcribed into cDNA by Reverse Transcription System (Vazyme, Nanjing, China). To quantify the mRNA levels of inflammatory factors, real-time PCR was performed using SYBRGreen Kit (Cwbio, Beijing, China) on a Bio-Rad CFX96 Detection System. The relative gene expression was normalized to β-actin. Data were analyzed by using the comparative Ct method. The primers of target genes used in this study were listed in Supplementary Table 1.

### Western Blot

The total protein from GC cells and MSCs were obtained with RIPA lysis buffer containing protease inhibitor cocktail (Invitrogen, United States). Proteins from each group were separated by SDS-polyacrylamide gel electrophoresis and then transferred onto polyvinylidene fluoride (PVDF) membranes. After blocking with 5% non-fat milk for 1 h, the membranes were incubated with primary antibodies at 4°C overnight. Primary antibodies including FAP (SAB, United States), α-SMA (BioWorld, United States), β-actin (SAB, United States), AKT/p-AKT (CST, United States), p38/p-p38 (CST, United States), STAT3/p-STAT3 (CST, United States), p65/p-p65 (CST, United States), ERK/p-ERK (CST, United States), N-cadherin (BioWorld, United States), E-cadherin (BioWorld, United States), Cyclin D1 (Abcam, United States), and PCNA (BioWorld, United States). The membranes were washed with TBST and incubated with secondary antibodies. The secondary antibodies were HRP-conjugated goat anti-rabbit and goat anti-mouse antibodies (SAB, United States). The bands were visualized by ECL reagent and the β-actin was served as the loading control.

### Animal Studies

All animal studies were performed by using 4–6 week female BALB/c nude mice (Model Animal Center of Nanjing University, Nanjing, China). GC cells (SGC-7901, 5 × 10^6^ cells in 100 μL PBS per mouse) pre-treated with different CMs for 24 h were subcutaneously injected into the flank of mice (*n* = 5/group). The volume (V) and weight of tumor were assessed every 5 days, and tumor volumes were calculated using the formula: *V* = 0.5 × *a* × *b*^2^, where *V* represents volume, *a* represents longitudinal diameter and *b* represents latitudinal diameter. To examine the metastatic ability of GC cells *in vivo*, the same number of GC cells was injected intraperitoneally into mice. All mice were killed at 4 weeks after injection, the metastases of tumor to different organs were observed and the number of visible metastatic nodules was recorded. The protocol was approved by the Animal Use and Care Committee of Jiangsu University (2014280).

### Immunohistochemistry

Tumor tissues were fixed with formaldehyde, embedded in paraffin and then cut into 5 μm sections. Briefly, the slides were dewaxed in xylene and rehydrated in graded ethanol solutions. Then, the sections were soaked in H_2_O_2_ to block endogenous peroxidase activity and autoclaved in a citrate buffer of pH6.0. The sections were incubated with primary monoclonal antibody against Ki-67 (CST, United States) followed by incubation with the secondary antibody for 30 min at room temperature and stained with H&E, respectively. The histological changes of tumor tissue were examined under an optical microscope (DP73; Olympus, Tokyo, Japan).

### Immunofluorescence

Immunofluorescence was performed to determine the expression of proteins related to CAFs markers. Cells were fixed with 4% paraformaldehyde for 10 min, permeabilized with 0.1% Triton X-100 for 10 min, then blocked with 5% bovine serum albumin, and incubated with goat anti-rabbit FAP (1:100, SAB, United States) and goat anti-mouse α-SMA antibody (1:150, BioWorld, United States) at 4°C overnight. The MSCs were then washed and incubated with Alexa Fluor 555 conjugated donkey anti-rabbit IgG or FITC conjugated goat anti-Rabbit IgG (Invitrogen, United States) for 1 h. The nuclei were stained with DAPI (1:200, Sigma-Aldrich) and images were acquired using a fluorescent microscope (Nikon, Japan). The experiments were repeated in triplicate for each group.

### Statistical Analysis

Statistical analyses were carried out by GraphPad Prism Software (version 4). All values were presented as mean values ± SD. Two-way ANOVA for multiple groups and unpaired Student’s *t* test for two groups were applied for statistical analysis. *P* < 0.05 was considered statistically significant.

## Results

### Neutrophils Treated With the Supernatant of Gastric Cancer Cells Stimulate the Transformation of MSCs to CAFs

**FIGURE 1:**
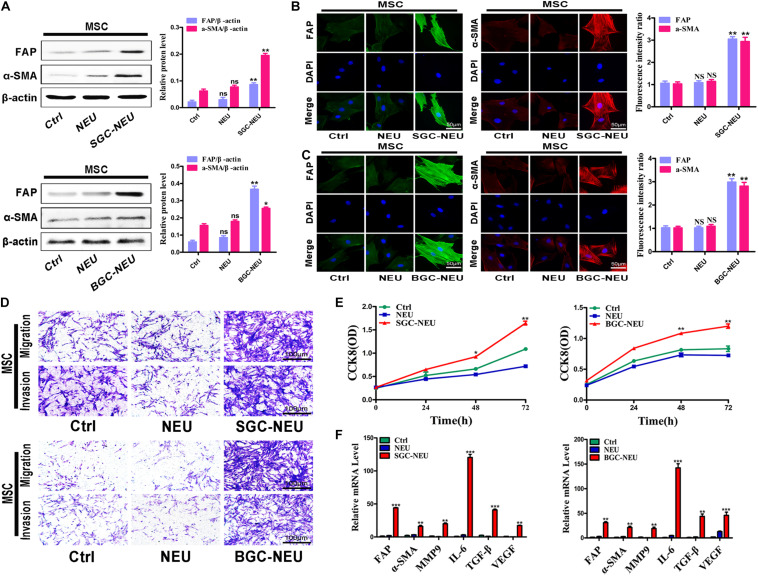
Tumor-educated neutrophils stimulate the transformation of MSCs to CAFs. **(A)** Western blot assays for the expression of FAP and α-SMA in MSCs treated with supernatant from neutrophils. **(B,C)** Immunofluorescent staining of FAP and α-SMA in MSCs after pretreated with different supernatant from SGC-NEU **(B)** and BGC-NEU **(C)**. **(D)** Cell migration/invasion assays for MSCs treated with supernatant from neutrophils. **(E)** CCK8 assay to detect the effect of supernatant from neutrophils on the proliferation of MSCs. **(F)** qRT-PCR analyses of the expression of FAP, α-SMA, MMP9, IL-6, TGF-β, and VEGF genes in MSCs treated with supernatant from neutrophils. Ctrl, MSCs; NEU, MSCs treated with CM of neutrophils; SGC/BGC-NEU, MSCs treated with CM from TENs. Magnification: 40×. (*n* = 3; *P < 0.05, **P < 0.01, and ***P < 0.001 compared to control group).

We first wanted to know whether TENs could activate MSCs. Human peripheral blood derived neutrophils were treated with the supernatant from GC cells and the CM of primed neutrophils was collected. After then, MSCs isolated from human umbilical cord were treated with TEN-CM for 24 h and the alterations in their phenotype and function were examined. The results of western blot showed that treatment with CM from TENs induced the expression of CAFs markers, including fibroblast activating protein (FAP) and α-smooth muscle actin (α-SMA), in MSCs (Figure 1A). Quantitative analysis of western blot also revealed an increase in FAP and α-SMA expression, in TEN-CM-treated MSCs (Figure 1A). Immunofluorescent staining and the statistical analysis of immunofluorescence intensity further demonstrated that TEN-CM stimulated the transformation of MSCs to CAFs (Figures 1B,C). Moreover, the migration and invasion capacities of MSCs were remarkably enhanced after incubation with TEN-CM (Figure 1D). We then performed CCK8 assay to determine the effect of TEN-CM on MSCs growth. As shown in Figure 1E, TEN-CM significantly promoted the proliferation of MSCs at 48 and 72 h after incubation. In addition, TEN-CM-treated MSCs showed increased expression of FAP, α-SMA, MMP9, IL-6, TGF-β, and VEGF genes (Figure 1F). Taken together, these results indicate that TEN-CM could induce MSCs to differentiate into CAFs and promote its proliferation and migration *in vitro*.

### Tumor-Educated Neutrophils Induce MSCs/CAFs Transformation Through AKT and p38 Signaling Pathways

**FIGURE 2:**
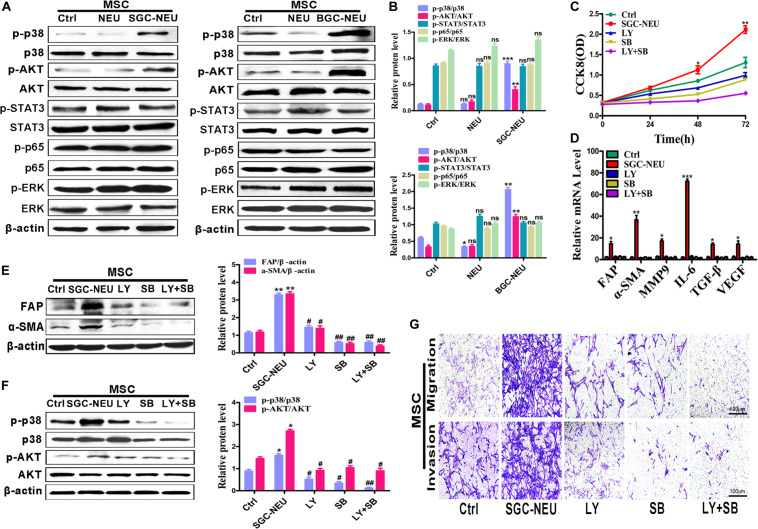
Tumor-educated neutrophils induce MSCs/CAFs transformation through AKT and p38 signaling pathways. **(A,B)** The expression of cell signaling pathway in MSCs treated with supernatant from neutrophils was determined by western blot **(A)** and the quantification of protein expression level **(B)**. **(C)** CCK8 assay to assess the proliferation of MSCs treated with supernatant from TENs in the presence or absence of AKT and p38 pathway inhibitors. **(D)** qRT-PCR analyses of the expression of FAP, α-SMA, MMP9, IL-6, TGF-β, and VEGF genes in MSCs treated with supernatant from TENs in the presence or absence of AKT and p38 pathway inhibitors. **(E)** MSCs were pre-treated with AKT and p38 pathway inhibitors followed by incubation with supernatant from TENs. The expression of FAP and α-SMA in MSCs was detected by western blot. **(F)** MSCs were pre-treated with AKT and p38 pathway inhibitors followed by incubation with supernatant from TENs. The expression of p-AKT and p-p38 in MSCs was detected by western blot. **(G)** MSCs were incubated with supernatant from TENs in the presence or absence of AKT and p38 pathway inhibitors and then used for cell migration/invasion assays. Ctrl, MSCs; NEU, MSCs treated with CM of neutrophils; SGC/BGC-NEU, MSCs treated with CM from TENs; LY, MSCs treated with CM from TENs in the presence of AKT pathway inhibitor (LY294002); SB, MSCs treated with supernatant from TENs in the presence of p38 pathway inhibitor (SB203580); LY + SB, MSCs pre-treated with AKT and p38 pathway inhibitors followed by incubation with CM from TENs. Magnification: 40×. (*n* = 3; *P < 0.05, **P < 0.01, and ***P < 0.001 compared to control group; ^##^*P* < 0.01 and ^#^*P* < 0.05 compared to SGC-NEU group).

To clarify the mechanism for MSCs activation by TENs, we treated MSCs with different TEN-CMs for 24 h and measured the responses of cell signaling pathway. TEN-CM treatment increased the expression of phosphorylated AKT (p-AKT) and p38 (p-p38) in MSCs and the statistical analysis of western blot (Figures 2A,B), indicating activation of AKT/p38 pathways. Moreover, pre-treatment with LY294002 and SB203580 restricted the increases in the proliferation abilities of MSCs after TEN-CM treatment (Figure 2C). In consistent with these observations, LY294002 and SB203580 almost completely blocked TEN-CM-induced expression of FAP, α-SMA, MMP9, IL-6, TGF-β, and VEGF genes in MSCs (Figure 2D). The induced expression of CAFs markers (FAP and α-SMA) in MSCs by TEN-CM was also reversed by specific inhibitors of AKT and p38 pathways (Figure 2E). Notably, pre-treatment with inhibitors of AKT (LY294002) and p38 (SB203580) pathways blocked the activation of AKT and p38 (Figure 2F) by TEN-CM. In addition, we also detected the effects of AKT (LY294002) and p38 (SB203580) pathway inhibitors on MSCs (control cells) alone. The pre-treatment with LY294002 and SB203580 shown no remarkable difference on the proliferation and the expression of inflammatory factor in MSCs (Supplementary Figures S1A,B). Consistent with that observation, LY294002 and SB203580 have no obvious impact on the expression of CAFs markers (FAP and α-SMA) and the activation of AKT and p38 in MSCs (Supplementary Figures S1C,D). Besides, the migration abilities of MSCs were inhibited after pretreated with LY294002 and SB203580 (Figure 2G). Taken together, these results indicate that TENs induce MSCs transformation to CAFs via activating AKT/p38 pathways.

### MSCs/CAFs Transformation Induced by Tumor-Educated Neutrophils Promotes the Proliferation, Migration, and Invasion of Gastric Cancer Cells *in vitro*

**FIGURE 3:**
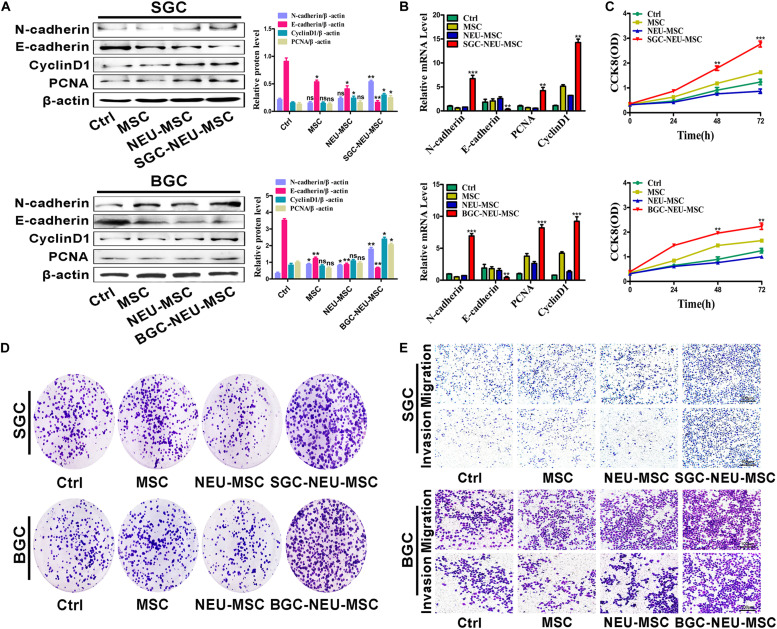
MSCs/CAFs transformation induced by gastric cancer-activated neutrophils promotes the proliferation, migration and invasion of gastric cancer cells *in vitro*. **(A)** Western blot assays for E-cadherin, N-cadherin, Cyclin D1, and PCNA expression in gastric cancer cells treated with different supernatant from neutrophils-activated MSCs. **(B)** The expression of E-cadherin, N-cadherin, Cyclin D1, and PCNA genes in gastric cancer cells treated with different supernatant from neutrophils-activated MSCs was determined by qRT-PCR. **(C)** The proliferation of gastric cancer cells treated with different supernatant from neutrophils-activated MSCs was detected by CCK8 assay. **(D)** Cell colony formation assays for gastric cancer cells that had been incubated with distinct supernatant from neutrophils-activated MSCs. **(E)** Transwell migration and matrigel invasion assays for gastric cancer cells treated with distinct supernatant from neutrophils-activated MSCs. Ctrl, SGC-7901/BGC-823; MSC, Gastric cancer cells treated with CM of MSCs; NEU-MSC, Gastric cancer cells treated with CM from neutrophils-primed MSCs; SGC/BGC-NEU-MSC, Gastric cancer cells pre-treated with the CM of MSCs activated by TENs. Magnification: 40×. (*n* = 3; *P < 0.05, **P < 0.01, and ***P < 0.001 compared to control group).

We then wanted to know the functional roles of MSCs/CAFs transformation induced by TENs. MSCs were treated with TEN-CM and the culture supernatant from MSCs was collected to act on GC cells for 24 h. We first tested the expression of proliferation and migration related proteins and genes in GC cells. We found that the expression of N-cadherin, Cyclin D1 and PCNA was increased, and the expression of E-cadherin was decreased in GC cells after treatment (Figures 3A,B). Then, the proliferation of GC cells was detected by CCK8 assay and cell colony formation assay. The proliferation abilities of GC cells treated with culture supernatant from TEN-CM-primed MSCs for 24 h were obviously higher than that of control cells (Figures 3C,D). The results of cell migration/invasion assays showed that the culture supernatant from TEN-CM-primed MSCs significantly increased the migration and invasion abilities of GC cells (Figure 3E). Collectively, these data indicate that TENs-induced MSCs/CAFs transformation promotes the proliferation, migration, and invasion of GC cells.

### Tumor-Educated Neutrophils Induce MSCs/CAFs Transformation Through the Release of Inflammatory Factors

**FIGURE 4:**
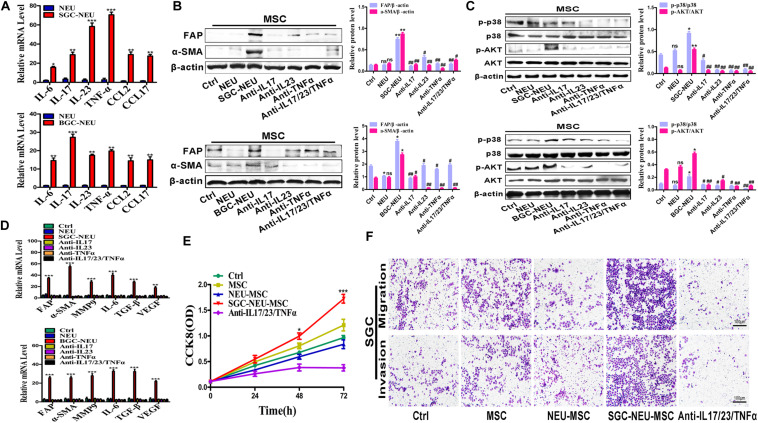
Tumor-educated neutrophils induce MSCs/CAFs transformation through the release of inflammatory factors. **(A)** The expression of inflammatory factor genes (IL-6, IL-17, IL-23, TNF-α, CCL2, and CCL17) in neutrophils was determined by qRT-PCR. **(B)** The expression of FAP and α-SMA in MSCs treated with supernatant from TENs in the presence or absence of neutralizing antibodies was determined by western blot. **(C)** The expression of p-AKT and p-p38 in MSCs treated with supernatant from TENs in the presence or absence of neutralizing antibodies was detected by western blot. **(D)** qRT-PCR analyses of the expression of FAP, α-SMA, MMP9, IL-6, TGF-β, and VEGF genes in MSCs treated with supernatant from TENs in the presence or absence of neutralizing antibodies. Ctrl, MSCs; NEU, MSCs treated with CM of neutrophils; SGC/BGC-NEU, MSCs treated with CM from TENs; Anti-IL17/23/TNFα, MSCs treated with supernatant from TENs in the presence of neutralizing antibodies. **(E)** CCK8 assay for the proliferation of gastric cancer cells treated with supernatant from TEN-CM-primed MSCs in the presence or absence of neutralizing antibodies. **(F)** Transwell migration and matrigel invasion assays for gastric cancer cells treated with supernatant from TEN-CM-primed MSCs in the presence or absence of neutralizing antibodies. Ctrl, SGC-7901 cells; MSC, SGC-7901 cells treated with CM of MSCs; NEU-MSC, SGC-7901 cells treated with CM from neutrophils-primed MSCs; SGC-NEU-MSC, SGC-7901 cells treated with CM of MSCs activated by TENs; Anti-IL17/23/TNFα, SGC-7901 cells treated with supernatant from TEN-CM-primed MSCs in the presence of neutralizing antibodies. Magnification: 40×. (*n* = 3; *P < 0.05, **P < 0.01, and ***P < 0.001 compared to control group; ^##^*P* < 0.01 and ^#^*P* < 0.05 compared to SGC/BGC-NEU group).

To explore which molecules released by TENs induce MSCs/CAFs transformation, we measured the expression of inflammatory factors in neutrophils after treatment with the supernatant from GC cells. Several genes of interest such as IL-17, IL-23, and TNF-α were identified to be highly expressed in the treated neutrophils (Figure 4A). We chose these three factors for the following studies due to their known relationships with pro-tumor roles of neutrophils and MSCs activation. Then, MSCs were treated with TEN-CM in the presence or absence of neutralizing antibodies against IL-17 (0.1 μg/mL), IL-23 (0.7 μg/mL) and TNF-α (0.05 μg/mL). Compared to SGC/BGC-NEU group, the expression of FAP and α-SMA proteins in neutralizing antibody group were significantly reduced (Figure 4B). The activation of AKT and p38 signaling pathway in MSCs by different TEN-CMs was significantly inhibited when neutralizing antibodies were added and the statistical analysis of western blot showed the same conclusion (Figure 4C). In addition, the expression of FAP, α-SMA, MMP9, IL-6, TGF-β, and VEGF genes in MSCs in neutralizing antibody group was also lower than that in SGC/BGC-NEU group (Figure 4D). Then, we detected the effects of anti-IL17, anti-IL23 and anti-TNFα on MSCs in control group. We found that there was almost no significant difference in the expression of FAP, α-SMA, MMP9, IL-6, TGF-β, and VEGF genes in MSCs between the groups (Supplementary Figure S2A). Compared to MSC group, the expression of CAFs markers and the activation of AKT and p38 signaling pathway in neutralizing antibody group had no remarkable difference (Supplementary Figures S2B,C). Moreover, the promotion of GC cell proliferation, migration and invasion abilities by culture supernatant from TEN-CM-primed MSCs was inhibited by the addition of neutralizing antibodies (Figures 4E,F). In summary, these data suggest that TENs induce MSCs/CAFs transformation through the release of IL-17/IL-23/TNF-α.

### Tumor-Educated Neutrophils Induce MSCs/CAFs Transformation to Promote Gastric Cancer Growth and Metastasis *in vivo*

**FIGURE 5:**
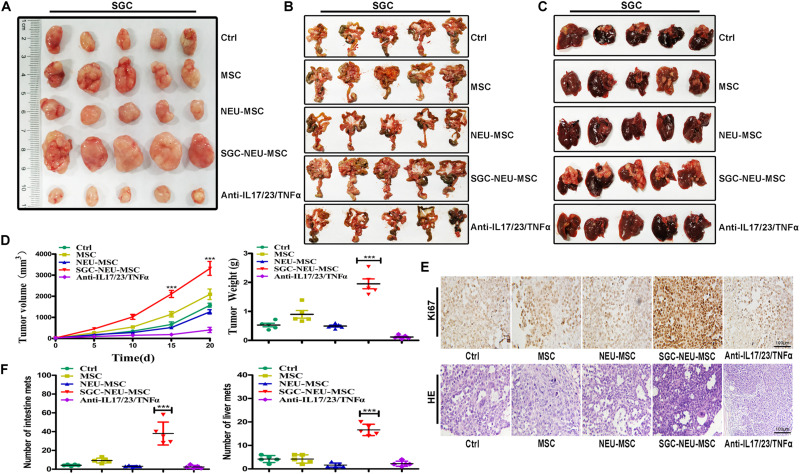
MSCs/CAFs transformation induced by tumor-educated neutrophils promotes gastric cancer growth and metastasis *in vivo*. **(A)** Representative images of tumor tissues in each group. **(B)** The metastatic tumor nodules in intestinal tissues of nude mice in each group. **(C)** The metastatic tumor nodules in livers of nude mice in each group. **(D)** Tumor volumes and weights of nude mice in each group (*n* = 5 per group). **(E)** Immunohistochemical staining of Ki-67 and H&E staining for mouse tumor tissues in different groups. **(F)** The number of metastatic tumor nodules in intestines and livers of nude mice in different groups. Ctrl, SGC-7901 cells; MSC, SGC-7901 cells treated with CM of MSCs; NEU-MSC, SGC-7901 cells treated with CM from neutrophils-primed MSCs; SGC-NEU-MSC, SGC-7901 cells pre-treated with the CM of MSCs activated by TENs; Anti-IL17/23/TNFα, SGC-7901 cells treated with supernatant from TEN-CM-primed MSCs in the presence of neutralizing antibodies. Magnification: 40×. (*n* = 5; **P < 0.01 and ***P < 0.001 compared to control group).

To further confirm the significance of MSCs/CAFs transformation induced by TENs in tumor progression, we treated GC cells for 24 h with culture supernatant from MSCs that had been primed with or without TEN-CM in the presence or absence of neutralizing antibodies. Then, GC cells were subcutaneously injected into BALB/c nude mice to establish xenograft tumor models. As shown in Figure 5A, significantly larger tumors were formed in mice injected with GC cells that had been treated with culture supernatant from TEN-CM-activated MSCs. However, the volume and weight of tumors formed by GC cells in neutralizing antibody group were remarkably smaller than that in TEN-CM-primed MSCs group (Figure 5D). Furthermore, the results of immunohistochemical analyses showed that tumor cells in TEN-CM-primed MSCs group had stronger Ki-67 staining than those in control group (Figure 5E). On the contrary, the positivity and intensity of Ki-67 staining in tumors was decreased in neutralizing antibodies group compared to TEN-CM-primed MSCs group (Figure 5E). To further investigate the metastatic potential of GC cells *in vivo*, we established mouse peritoneal metastasis model. We found that treatment with culture supernatant from TEN-CM-primed MSCs promoted the colonization of GC cells in the intestines (Figure 5B) and enhanced the capacity of GC cells to metastasize to the livers (Figure 5C). The number of visible metastatic nodules was recorded and analyzed statistically. Consistent with that observation, the number of tumor nodules in the intestines and livers of mice in TEN-CM-primed MSCs group were more than that in control and unprimed MSCs groups (Figure 5F). However, the number of tumor nodules in the intestines and livers of mice in neutralizing antibody group was less than that in TEN-CM-primed MSCs group (Figure 5F). In conclusion, these findings suggest that TENs induce MSCs/CAFs transformation to promote GC growth and metastasis *in vivo*.

## Discussion

**FIGURE 6:**
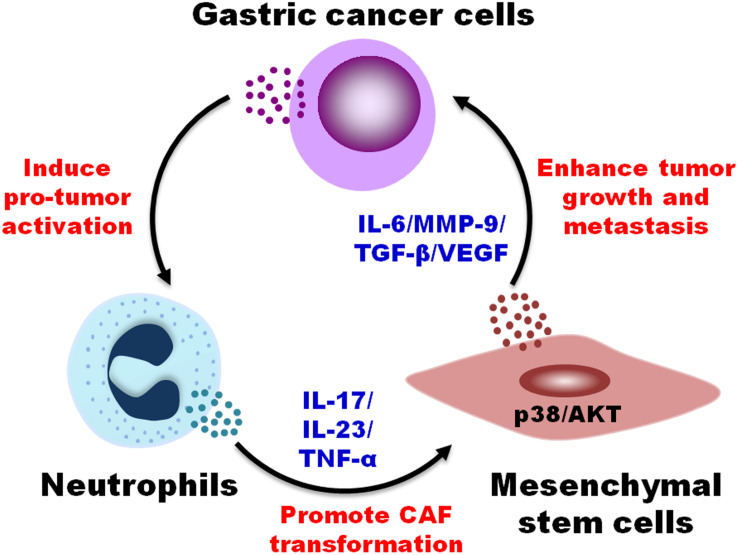
A proposed model for the regulation of MSCs/CAFs transformation by tumor-educated neutrophils in gastric cancer progression. Neutrophils are educated by gastric cancer cells in tumor microenvironment to acquire a pro-tumor phenotype and function. Tumor-educated neutrophils induce the transformation of MSCs to CAFs through the production of inflammatory factors (i.e., IL-17, IL-23, and TNF-α) and the activation of AKT and p38 pathways, which in turn promotes the proliferation, migration, and invasion of gastric cancer cells, finally accelerating gastric cancer growth and metastasis.

In this study, we reported that TENs induced MSCs/CAFs transformation to promote GC progression. TENs derived factors trigged the differentiation of MSCs to CAFs by activating AKT/p38 pathways. In turn, TENs-activated MSCs accelerated GC growth and metastasis both *in vitro* and *in vivo*. When the neutralizing antibodies of IL-17, IL-23, and TNF-α were added before priming, the transformation of MSCs to CAFs by TENs was blocked. Then, the promoting roles of MSCs in tumor growth and metastasis were also greatly reduced (Figure 6). The findings of our study provide new evidence for the pro-tumor roles of neutrophils in cancer and reveal a novel mechanism for MSCs regulation in GC.

Cancer-associated fibroblasts are one of the key components of TME and are critically involved in tumor progression (Chen and Song, 2019; Kobayashi et al., 2019). Accumulating studies indicate that cancer development depends not only on malignant cancer cells, but also on stromal activation (Quante et al., 2011). The interplay between CAFs and cancer cells is critical for cancer cell proliferation, invasion, metastasis, and other malignant biological behaviors. We have previously shown that neutrophils educated by GC cells displayed pro-tumor phenotype and function (Zhang et al., 2018). TENs could promote the migration and invasion of GC cells through direct induction of EMT (Zhang et al., 2017). However, whether TENs could regulate CAFs are not clear. In this study, we aimed to investigate whether TENs could induce MSCs activation to CAFs. The roles of CAFs and TENs in cancer have been firmly established (Coffelt et al., 2016; Chen and Song, 2019). In this study we reported that TENs could induce the transformation of MSCs into CAFs, while activated MSCs in turn promote the proliferation, migration and invasion of GC cells. These data suggest that there may be a positive feedback loop among cancer cells, neutrophils, and MSCs that synergistically drives GC progression, which provides new evidence for the important role of inflammation in cancer.

Neutrophils have been reported to recruit regulatory T cells and macrophages to promote the progression of hepatocellular carcinoma and resistance to sorafenib (Zhou et al., 2016). Wang et al. (2017) suggest that neutrophils could inhibit T cell function through PD-L1/PD-1 interaction to promote GC growth, suggesting that neutrophils can regulate other non-tumor cells in TME to promote tumor progression. In the current study, we showed that TENs could induce the MSCs/CAFs switch, which further enhanced the malignant behaviors of GC cells, suggesting that TENs could reprogram MSCs to create a favorable microenvironment for tumor progression.

In human cancers, neutrophils exert tumor-promoting functions by producing a variety of factors, including HGF, CCL17, BMP2, among others (Coffelt et al., 2016; Shaul and Fridlender, 2019; Zhou et al., 2019). Our data showed that TENs induced MSCs/CAFs transformation by producing IL-17, IL-23, and TNF-α. Li et al. (2017) demonstrate that IL-17 is primarily expressed by neutrophils in GC tissues. They also found that neutrophils were enriched predominantly in the invasive margin, and neutrophil levels were a powerful predictor of poor survival in patients with GC (Li et al., 2017). Clinical studies have suggested that elevated numbers of IL-17-producing cells infiltrating in tumors are an independent marker of adverse prognosis in patients with cancer (Chung et al., 2013). IL-17 stimulates GC cells to express CXC chemokines, which recruits neutrophils into GC tissue to promote angiogenesis. Another study suggests that neutrophils derived IL-17 induce EMT in GC cells to promote their migration and invasion (Li et al., 2019). In addition, neutrophils infiltrated in the colon tissues of patients with IBD have been reported to be the main source of IL-23 and increased IL-23 expression is associated with the pathogenesis of IBD (Kvedaraite et al., 2016). Intriguingly, IL-23, when combined with IL-6, could induce IL-17 expression in neutrophils in GC (Li et al., 2017). Increased TNF-α expression in neutrophils has also been reported in late-stage tumor mouse model and neutrophils induce apoptosis of CD8^+^ T cells through TNF-α (Mishalian et al., 2013; Michaeli et al., 2017). We found that when IL-17/IL-23/TNF-α were neutralized, TENs failed to induce MSCs/CAFs transformation and the effects of TENs-activated MSCs on promoting GC growth and metastasis were significantly attenuated, indicating that these factors are important for TENs-induced MSCs/CAFs transformation. The secreted factors that lead to tumor cells activation of the neutrophils have been widely reported, including CXCL5, HGF, OSM, HA, among others (Zhou et al., 2016, 2019; Liang et al., 2018). Recently, we reported that GC cells derived exosomal HMGB1 could activate neutrophils (Zhang et al., 2018). We have previously shown that MSCs resident in GC tissues could induce pro-tumor activation of neutrophils via IL-6 (Zhu et al., 2014). Yu et al. (2017) have shown that TNFα-activated mesenchymal stromal cells promote breast cancer metastasis by recruiting CXCR2^+^ neutrophils. These findings, when combined together, suggest that a bidirectional crosstalk may exist between neutrophils and MSCs in TME and that neutrophils cooperate with MSCs to promote cancer progression.

Recent studies demonstrate that inflammatory factors play an active role in tumor growth and progression (Viola et al., 2012). Tumor-infiltrating neutrophils in melanoma are associated with poor outcome (Jensen et al., 2012). In addition, high densities of neutrophils in tumor are identified as an independent risk factor for poor prognosis (Shen et al., 2014). Several inflammatory factors have been previously shown to be involved in AKT/p38 signaling pathway activation and MSCs/CAFs transformation (Ridge et al., 2017; Timaner et al., 2020). Our observations showed that TENs induced MSCs/CAFs transformation by activating AKT/p38 pathways. When the activation of AKT/p38 pathways was inhibited by specific inhibitors, TENs-induced CAFs transformation and pro-tumor functions in MSCs were reversed, suggesting a key role of these pathways in MSCs/CAFs transformation in this setting. CAFs can secrete a variety of factors, including TGF-β, MMPs and interleukins, to promote EMT of cancer cells and stimulate the invasive and metastatic potential of cancer cells (Chen and Song, 2019; Kobayashi et al., 2019). Herein, we found that TENs-activated MSCs expressed higher levels of IL-6, MMP9, TGF-β, and VEGF, which have been previously shown to be associated with the pro-tumor functions of MSCs (McAndrews et al., 2015; Scherzad et al., 2015; Ridge et al., 2017; Timaner et al., 2020). Shrestha et al. (2016) demonstrate that neutrophils activate MSCs in an ERK-dependent manner and neutrophils-activated MSCs enhance the growth of germinal center B-cell lymphoma cells more efficiently. In this study, we measured the expression of cell signaling pathway in MSCs treated with supernatant from neutrophils. We found that TENs induce MSCs transformation to CAFs via activating AKT/p38 pathways. These findings suggest that neutrophils may regulate MSCs activation and function through distinct mechanisms in different cancers.

## Conclusion

In summary, our study suggests that TENs are able to induce MSCs transition to CAFs via increased expression of inflammatory factors IL-17/IL-23/TNF-α and the subsequent activation of AKT and p38 signaling pathways, which finally leads to enhanced GC growth and metastasis. These findings provide mechanistic insights into the regulation of MSCs by neutrophils in GC as well as offer novel strategies for the design of new therapeutic strategies.

## Data Availability Statement

The raw data supporting the conclusions of this article will be made available by the authors, without undue reservation, to any qualified researcher.

## Ethics Statement

The animal study was reviewed and approved by University Committee on Use and Care of Animals of Jiangsu University (2012258).

## Author Contributions

JZ, CJ, and HS completed all the experiments and wrote the manuscript. WX, HQ, and XZ designed the outline of the experiments. WL, ZM, YS, and RJ reviewed and corrected the draft. All authors contributed to the article and approved the submitted version.

## Conflict of Interest

The authors declare that the research was conducted in the absence of any commercial or financial relationships that could be construed as a potential conflict of interest.

## Supplementary Material

FIGURE S1 Effects of AKT and p38 pathway inhibitors on MSCs. **(A)** CCK8 assay for the proliferation of MSCs treated with LY294002 and SB203580. **(B)** qRT-PCR analyses of the expression of FAP, α-SMA, MMP9, IL-6, TGF-β, and VEGF genes in MSCs treated with LY294002 and SB203580. **(C)** The expression of FAP and α-SMA in MSCs treated with LY294002 and SB203580 was detected by western blot. **(D)** Western blot assay for the expression of p-AKT and p-p38 in MSCs pre-treated with LY294002 and SB203580.

FIGURE S2 Detecting the effects of anti-IL17, anti-IL23 and anti-TNFα on MSCs. **(A)** The expression of FAP, α-SMA, MMP9, IL-6, TGF-β, and VEGF genes in MSCs treated with neutralizing antibodies was detected by qRT-PCR. **(B)** Western blot assays for the expression of FAP and α-SMA in MSCs treated with neutralizing antibodies. **(C)** The expression of p-AKT and p-p38 in MSCs treated with neutralizing antibodies was detected by western blot.
