# Supplementary figures and images for: Tumor-Educated Neutrophils Activate Mesenchymal Stem Cells to Promote Gastric Cancer Growth and Metastasis
Source: Front Cell Dev Biol. 2020 Aug 13;8:788. doi: 10.3389/fcell.2020.00788 (PMC7438587; doi:10.3389/fcell.2020.00788)

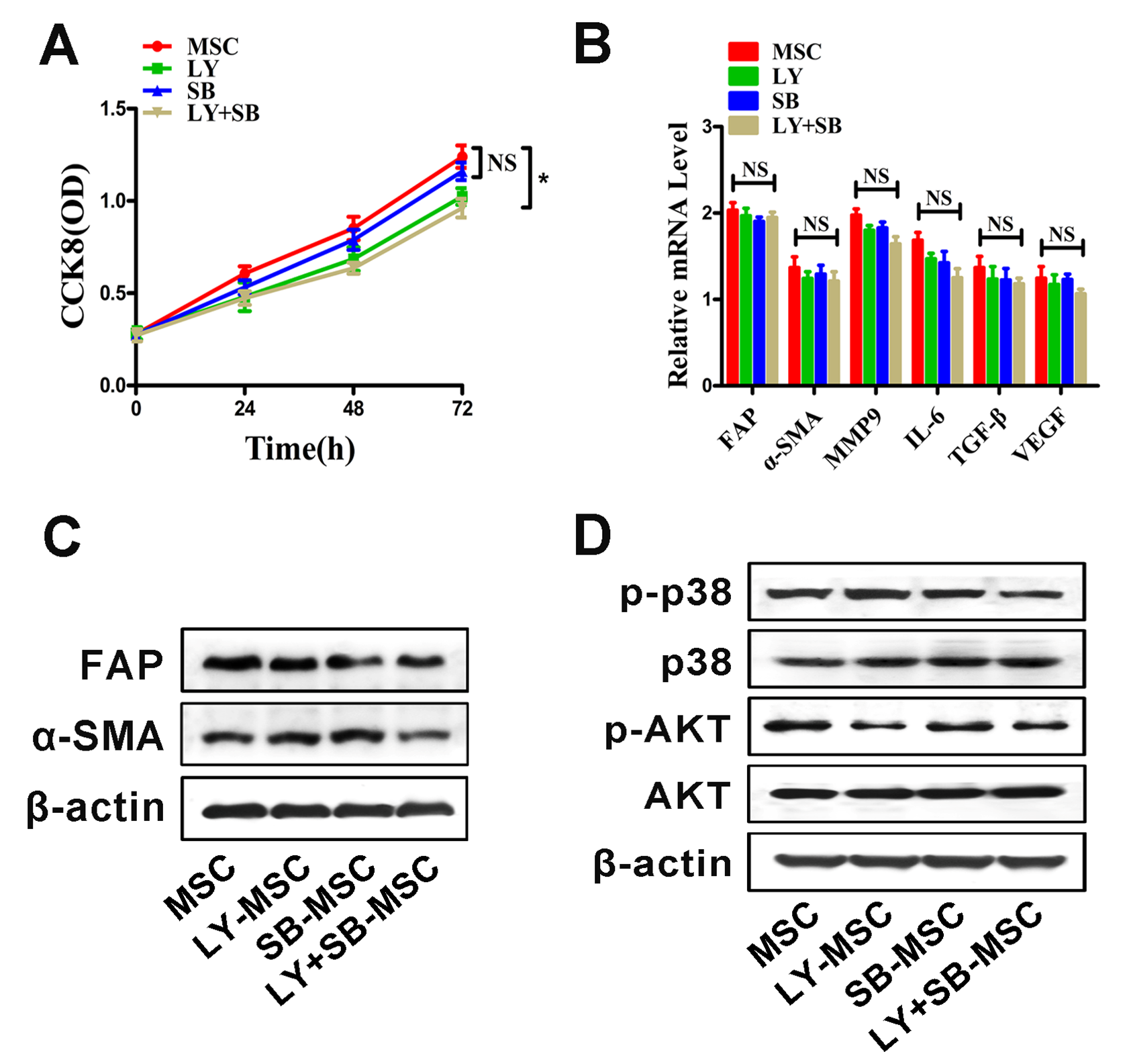

Supplement: FIGURE S1 — Effects of AKT and p38 pathway inhibitors on MSCs. (A) CCK8 assay for the proliferation of MSCs treated with LY294002 and SB203580. (B) qRT-PCR analyses of the expression of FAP, α-SMA, MMP9, IL-6, TGF-β, and VEGF genes in MSCs treated with LY294002 and SB203580. (C) The expression of FAP and α-SMA in MSCs treated with LY294002 and SB203580 was detected by western blot. (D) Western blot assay for the expression of p-AKT and p-p38 in MSCs pre-treated with LY294002 and SB203580. [file Image_1.tif]

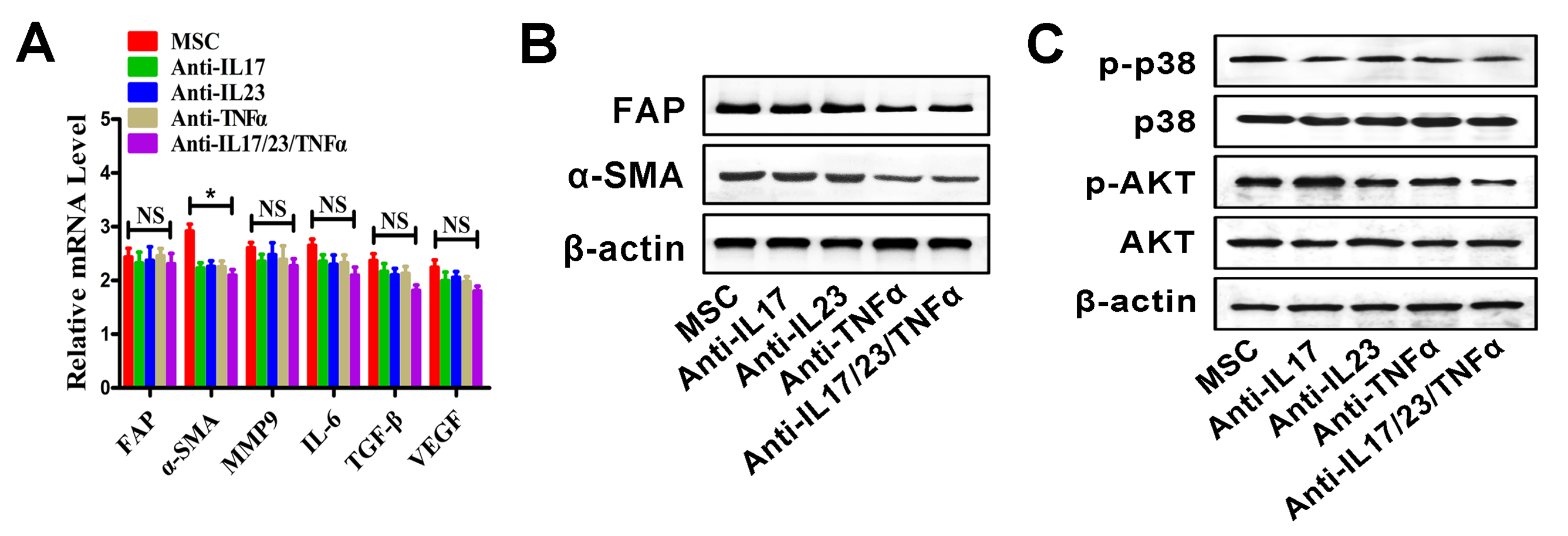

Supplement: FIGURE S2 — Detecting the effects of anti-IL17, anti-IL23 and anti-TNFα on MSCs. (A) The expression of FAP, α-SMA, MMP9, IL-6, TGF-β, and VEGF genes in MSCs treated with neutralizing antibodies was detected by qRT-PCR. (B) Western blot assays for the expression of FAP and α-SMA in MSCs treated with neutralizing antibodies. (C) The expression of p-AKT and p-p38 in MSCs treated with neutralizing antibodies was detected by western blot. [file Image_2.tif]
