# Supplementary material for: Tumor-Educated Neutrophils Activate Mesenchymal Stem Cells to Promote Gastric Cancer Growth and Metastasis
Source: Front Cell Dev Biol. 2020 Aug 13;8:788. doi: 10.3389/fcell.2020.00788 (PMC7438587; doi:10.3389/fcell.2020.00788)
Supplement: Supplementary file 3 [file Table_1.docx]

**Table 1. The sequences of primers for target genes.**

| Gene | Sequence | Size (bp) | T_m_ (^o^C) |
| --- | --- | --- | --- |
| β-actin | F:5'-CACGAAACTACCTTCAACTCC-3'  R: 5'-CATACTCCTGCTTGCTGATC-3' | 265 | 60 |
| IL-6 | F: 5'-TACATCCTCGACGGCATCTC-3'  R: 5'-AGCTCTGGCTTGTTCCTCAC-3' | 252 | 61 |
| TNFα | F: 5'-CCGAGTGACAAGCCTGTAGC-3'  R: 5'-AGGAGGTTGACCTTGGTCTG-3' | 493 | 57 |
| E-cadherin | F:5’-CGCATTGCCACATACACTCT-3’  R:5’-TTGGCTGAGGATGGTGTAAG-3’ | 252 | 55 |
| N-cadherin | F:5’-AGTCAACTGCAACCGTGTCT-3’  R:5’-AGCGTTCCTGTTCCACTCAT-3’ | 337 | 55 |
| Cyclin D1 | F: 5'-CCGAGAAGCTGTGCATCTAC-3'  R:5'-CTTCACATCTGTGGCACAGAG-3' | 221 | 55 |
| PCNA | F:5'-TTTGGTGCAGCTCACCCTG -3'  R:5'-CGCGTTATCTTCGGCCCTTA -3' | 151 | 60 |
| α-SMA | F: 5'- CTGACTGAGCGTGGCTATTC -3'  R:5'- CCACCGATCCAGACAGAGTA -3' | 452 | 58 |
| FAP | F: 5'- ATAGCAGTGGCTCCAGTCTC -3'  R:5'-GATAAGCCGTGGTTCTGGTC -3' | 278 | 59 |
| MMP9 | F: 5'-ACGTCTTCCAGTACCGAGAG -3'  R:5'- GGCACTGCAGGATGTCATAG -3' | 126 | 60 |
| VEGF | F:5'-CCTTGCTGCTCTACCTCCAC-3'  R:5'-ATCTGCATGGTGATGTTGGA-3' | 280 | 61 |
| TGF-β | F: 5'-CACACTGCAAGTGGACATC -3'  R:5'-GCAGAAGTTGGCATGGTAG-3' | 277 | 55 |
| IL-17 | F:5'-GGTCTTCATTGCGGTGGA -3'  R:5'-GAAGGCAGCAGCGATCAT -3' | 185 | 67 |
| IL-23 | F:5'-GGACAACAGTCAGTTCTGCTTGC-3'  R:5'-GGAGGCTGCGAAGGATTTTG-3' | 200 | 65 |
| CCL2 | F: 5'-GAACCGAGAGGCTGAGACTA-3'  R: 5'-GCCTCTGCACTGAGATCTTC-3' | 151 | 62 |
| CCL17 | F: 5'- AGTGTCACCGCCTGCTGAT-3'  R: 5'- CGTGGATGTGCTGCAGAGAA-3' | 168 | 63 |
